# Synergies of Targeting Tumor Angiogenesis and Immune Checkpoints in Non-Small Cell Lung Cancer and Renal Cell Cancer: From Basic Concepts to Clinical Reality

**DOI:** 10.3390/ijms18112291

**Published:** 2017-10-31

**Authors:** Andreas Pircher, Dominik Wolf, Axel Heidenreich, Wolfgang Hilbe, Renate Pichler, Isabel Heidegger

**Affiliations:** 1 Internal Medicine 5, Department of Hematology and Oncology, Medical University Innsbruck, Anichstreet 35, 6020 Innsbruck, Austria; 2 Medical Clinic 3, Department of Oncology, Hematology, Immunoncology and Rheumatology, University Hospital Bonn (UKB), 53127 Bonn, Germany; dominik.wolf@ukbonn.de; 3 Department of Urology, Uro-Oncology, Robot-Assisted and Reconstructive Urologic Surgery, University Hospital Cologne, 50937 Cologne, Germany; axel.heidenreich@uk-koeln.de; 4 Department of Internal Medicine I, Center for Oncology, Hematology and Palliative Care, Wilhelminenspital, 1160 Vienna, Austria; wolfgang.hilbe@wienkav.at; 5 Department of Urology, Medical University Innsbruck, 6020 Innsbruck, Austria; renate.pichler@i-med.ac.at (R.P.); isabel-maria.heidegger@i-med.ac.at (I.H.)

**Keywords:** angiogenesis, immunotherapy, checkpoint inhibition, VEGF inhibition, renal cell cancer, non-small cell lung cancer

## Abstract

In recent years, considerable advances concerning therapeutic strategies in patients with metastatic cancer have been achieved. Particularly in renal cell cancer (RCC) and advanced stage non-small cell lung cancer (NSCLC), immune-activating and antiangiogenic (AA) drugs (i.e., checkpoint antibodies and vascular endothelial growth factor (VEGF)/VEGF receptors (VEGFR) targeting compounds, respectively) have been successfully developed. As immune-effector cells have to enter the tumor, it is tempting to speculate that the combination of immunotherapy with AA treatment may induce synergistic effects. In this short review, we explore the theoretical background and the therapeutic potential of this novel treatment option for patients with advanced RCC or NSCLC. We discuss the growing body of evidence that pro-angiogenic factors negatively modulate the T-cell-mediated immune response and examine the preclinical evidence for testing combined immune-activating and AA therapy concepts in clinical practice. Particular attention will also be paid to potential novel treatment-related adverse events induced by combination treatment.

## 1. Introduction

**Figure 1:**
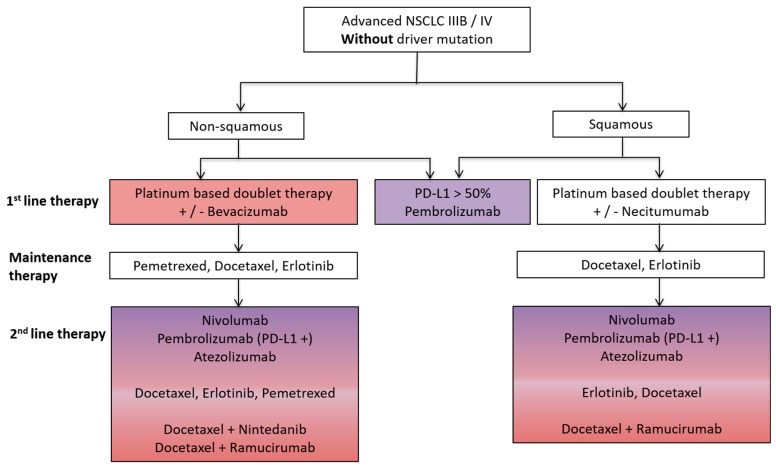
Schematic overview of a standard treatment algorithm in patients with advanced non-small cell lung cancer (NSCLC) IIIB/IV without driver mutation according to the European society of medical oncology (ESMO) guidelines 2016 [1]. Pink: antiangiogenic agent approved as a treatment option; Purple: immunotherapy approved as a treatment option; Pink/Purple: both antiangiogenic agent and immunotherapy approved as a treatment option. PD-L1, programmed death ligand-1.

**Figure 2:**
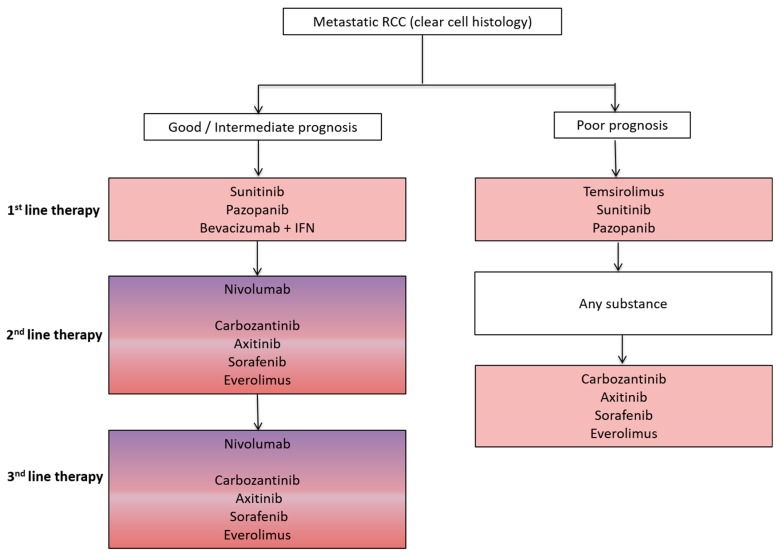
Schematic overview of a standard treatment algorithm in patients with metastatic clear cell renal cell cancer (RCC) according to the 2017 European association of urology (EAU) guidelines (Available online: www.uroweb.org, accessed on 29 October 2017). For detailed treatment information including level of evidence and a treatment algorithm based on previous therapies, we refer to the EAU guidelines. Pink: antiangiogenic agent approved as a treatment option; Pink/Purple: both antiangiogenic agent and immunotherapy approved as a treatment option. IFN, interferon.

Advanced stage non-small cell lung cancer (NSCLC) and renal cell cancer (RCC) represent prototypes of solid tumors responsive to both antiangiogenic (AA) and immunotherapy. Both AA and immunotherapy are guideline-based therapeutic options for both NSCLC and RCC (Figure 1 and Figure 2) due to their excellent treatment responses resulting in increased overall survival (OS) and progression free survival (PFS) rates in both tumor entities.

Recent preclinical studies strongly suggest that AA agents synergistically act together with immune-activating drugs in metastatic cancer [2]. Therefore, it is of high interest to further explore the potential synergistic activity of AA compounds, which induce vascular normalization therapy potentially improving cancer infiltration by immune effector cells. Vice versa, the impact of activated immune cells on the tumor vasculature should be defined in more detail. 

Based on these observations, several studies in both NSCLC and RCC patients have been initiated investigating the therapeutic effect as well as potential treatment-related adverse events of combined AA and immunotherapy.

## 2. Preclinical Rationale for Combined AA and Immune-Activating Therapy

There is growing evidence that the tumor microenvironment (TME) is crucially involved in cancer development and progression [3]. In general, the TME is composed of two different cell types: (i) cells that are present in the normal tissue before tumor development (endothelial cells, fibroblasts) and (ii) cells that are hired to the TME (mainly bone marrow derived immune cells). Key strategies of targeting the TME are the inhibition of angiogenesis as well as the stimulation of an effective immune response, both hallmarks of cancer [4,5].

Blood vessel formation in malignant tumors is mainly governed by hypoxia-driven over-production of the vascular endothelial growth factor (VEGF) [6]. This led to successful implementation of several therapies targeting VEGF itself or its signaling via the VEGF receptors (VEGFR) [7]. In addition, targeting the immune system was revolutionized by the discovery of immune checkpoints which limits the endogenous anti-tumor immune response, finally leading to immune evasion of cancer [8]. Successful reversal of this immune evasion can be achieved by checkpoint inhibitors, particularly in cancers with a high immunogenic potential (e.g., characterized by a high mutational burden). Targeting the cytotoxic T lymphocyte- associated protein-4 (CTLA-4), programed cell death protein-1 (PD-1) or programed death ligand-1 (PD-L1) by inhibitory antibodies is approved for various cancer types including NSCLC and RCC. In both diseases, those drugs may induce long-lasing remissions with OS prolongation [9]. Of note, the PD-1-targeting monoclonal antibody (mAb) pembrolizumab has recently even been approved to replace chemotherapy in the first-line therapy of NSCLC with high expression of PD-L1 [10].

Blood vessel tortuosity in tumors hampers the accessibility and homing of immune cells. A possible concept to circumvent these limitations is to normalize the tumor vasculature by restoring a balance of pro- and antiangiogenic factors, thereby inducing a more hostile microenvironment actively stimulating immune activation [11,12]. For example, in breast cancer, it has been previously shown that high microvascluar density (MVD) at therapy start predicts bevacizumab (anti-VEGF mAB) efficacy; moreover, patients with low MVD did not benefit from the AA therapy [13]. Summarizing, this study gives a strong hint that (i) AA therapies need an adequate number of preexisting vessels to achieve a vascular normalization phenotype and (ii) that tumors with low MVD tend to form immature vessels, resulting in an insufficient number of functional vessels [13]. 

Interestingly, it has been shown that AA agents are able to stimulate the immune system via induction of a more hostile tumor microenvironment for an effective anti-tumor immune reaction [14,15,16]. On the other hand, checkpoint inhibitors lead to vascular normalization via the induction of type 1 T-helper (TH-1) cells in the TME, which have been shown to co-localize with tumor endothelial cells and induce changes in the cytokine environment, and subsequently affect pericyte recruitment/attachment [17]. Furthermore, the vessel normalization leads to changes in the immune cell composition governed by recruiting T lymphocytes and decreasing neutrophils. This process and the regulatory feedback loop are modulated in part by interferon γ (IFNγ) and CD40 ligand (CD40L) [17]. However, to date, the biological background of the complex and dynamic interactions of targeting tumor angiogenesis and inducing anti-tumor immune response have not been sufficiently investigated [18]. 

The tumor vasculature is the gate for immune cell infiltration into the tumor and may thus also be critical for cancer immune evasion by preventing sufficient immune cell access to the tumor and its microenvironment. In addition, endothelial cells (ECs) are also involved in immune-regulation by presenting antigens via the expression of major histocompatibility complex class I, class II, as well as co-stimulatory molecules such as CD80 and CD86, and the secretion of immunosuppressive cytokines [3,19,20]. Another important function of ECs is the regulation of immune cell trafficking into the tumor tissue by expression of vascular adhesion molecules [3]. According to the idea that the prevention of immune cell infiltration is an immune evasion mechanism, tumors with higher infiltration of tumor infiltrating lymphocytes (TILs) have a better prognosis, and inflammatory cytokines such as tumor necrosis factor (TNF)-α, interleukin (IL)-1 and IFNγ regulate EC adhesion molecule expression, thus further controlling immune cell infiltration [3]. However, endothelial anergy, describing that ECs are not responsive to the cytokine milieu generated by the TME, may counteract the successful recruitment of immune effector cells. EC anergy is mainly mediated by the hypoxic microenvironment associated with the overexpression of immunosuppressive cytokines including VEGF as well as an acidic milieu dampening successful immune activation [3]. Moreover, excessive production of VEGF in the TME also promotes immune-inhibitory cells, such as regulatory T cells (Tregs) and myeloid-derived suppressor cells (MDSCs) and tolerogenic dendritic cells (DCs), which explains, at least in part, the vicious circle of tumor angiogenesis and immunosuppression [21,22]. Interestingly, there is evidence that AA therapies can reverse EC anergy and induce adhesion molecule expression, facilitating immune infiltration [23], highlighting the complex interplay between tumor angiogenesis and anti-tumor immunity. In addition to the ECs themselves, the overall tumor vasculature is also known to be chaotic, torturous and characterized by hyperactive EC [11], which leads to loose connections between ECs and high interstitial pressure, again impeding immune cell trafficking [24]. Tumor vessels are also less covered with pericytes, which further increases vascular permeability, finally also contributing to metastasis formation [11,12]. 

**Figure 3:**
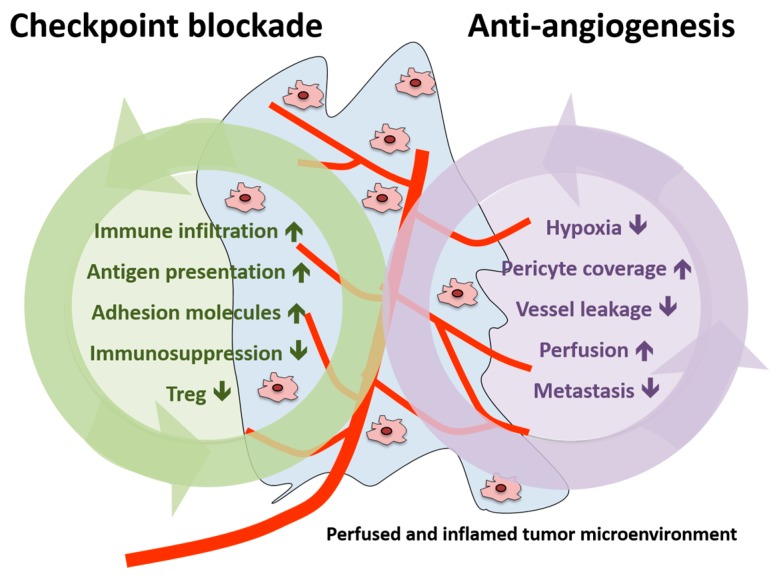
Schematic presentation of the effects of immune checkpoint blockade and antiangiogenesis in both the tumor and the tumor microenvironment. Treg, regulatory T cell.

In summary, vascular normalization strategies were shown to reduce tumor hypoxia, vessel leakage and metastasis formation in the preclinical setting as well as to increase pericyte coverage and tumor blood perfusion [25]. Therefore, the addition of AA agents to immunotherapy could potentiate the effects induced by checkpoint inhibition (increase in immune infiltration, antigen presentation, adhesion molecules expression and reduction in immunosuppression and Treg) or act in a synergistic manner (summarized in Figure 3).

## 3. Non-Small Cell Lung Cancer (NSCLC)

Lung cancer is the leading cause of cancer-related death in the United States and NSCLC is the most common type of disease [26]. For several years, AA agents such as bevacizumab have been established as standard treatment options for adeno-carcinoma of the lung. Moreover, in recent years, immunotherapeutic drugs such as nivolumab, pembrolizumab (targeting PD-1) or atezolizumab (targeting PD-L1) have been approved as standard therapeutic treatment options in NSCLC depending on the tumor’s molecular subtype [1,27]. An overview of the basic standard guidelines for the treatment of NSCLC is outlined in Figure 1. 

However, even though both therapy concepts have clearly advanced the field, many patients are still primary refractory or relapse/progress upon excellent initial treatment response to standard therapy. Thus, a high medical need for novel innovative treatment concepts including combination therapies is clearly warranted.

## 4. Preclinical Concepts Combining AA and Immunotherapies in NSCLC

It has recently been shown in a preclinical study that the VEGF inhibitor bevacizumab can improve the antitumor efficacy of cytokine-induced killer (CIK) cells transfer therapy, thereby pointing out that combining AA therapy with CIK cells may enhance therapeutic efficacy in NSCLC [28]. A similar study was also able to demonstrate that recombinant human endostatin improves the treatment effect of adoptive CIK cells therapy against lung carcinomas, thereby unmasking the mechanism of the synergistic antitumor efficacy using murine models [29].

At the American society of clinical oncology (ASCO) meeting in 2017, a Japanese research group reported novel data on the combination of AA (sunitinib) and an antitumor immunotherapy using an agonistic antibody against the death receptor-5 in mice. The tyrosine kinase inhibitor (TKI) sunitinib increased lymphatic flow and decreased hypoxic areas in the tumor. In addition, the combination therapy also decreased tumor growth rates compared to monotherapy. Thus, combination treatment increased the number of CD4^+^FOXP3^−^ and CD8^+^ T cells in tumors compared to controls. For underlying the immune-mediated effect of combinational treatment, the authors conducted CD4^+^ and/or CD8^+^ T cell depletion studies, which partially proved CD8^+^ T cell dependency in combinational treatment.

These findings support the idea that blood vessel normalization and improved lymphatic flow in tumors improves the circulation between the tumors and draining lymph nodes, thereby augmenting the effects of immunotherapy [30].

**Figure 4:**
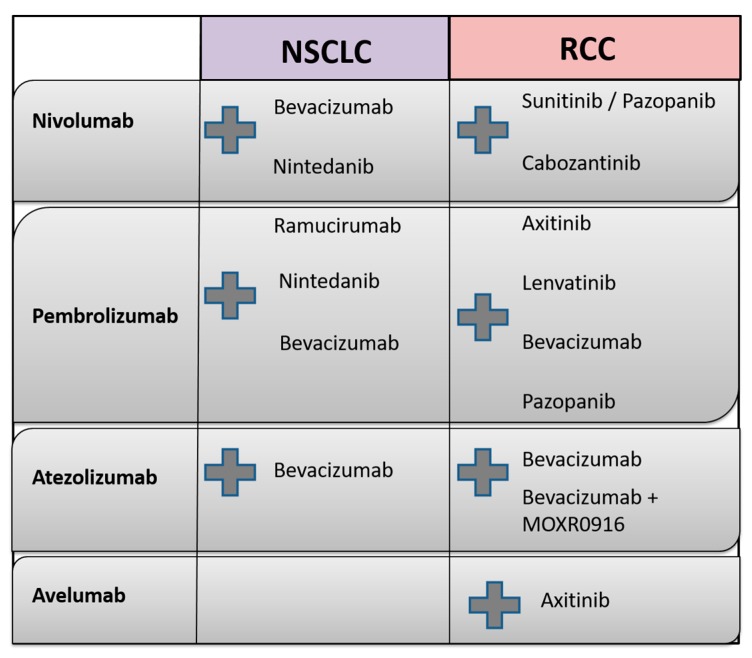
Overview of combination treatment of immune checkpoint inhibitors (nivolumab, pembrolizumab, avelumab and atezolizumab) with antiangiogenic agents currently investigated in clinical studies in both NSCLC and RCC (the combination treatment is represented by the grey plus sign).

Currently, several studies combining AA with checkpoint blockade are actively recruiting patients; these studies are discussed in detail in the following. An overview of the molecular targets of agents used in clinical NSCLC and RCC studies and ongoing treatment combinations of AA and immunotherapeutic agents is outlined in Table 1, as well as in Figure 4, respectively.

## 5. Clinical Studies Combining AA and Immunotherapies in NSCLC

### 5.1. Clinical Studies Combining Bevacizumab with Nivolumab

The impact of maintenance nivolumab therapy combined with bevacizumab vs. nivolumab monotherapy was evaluated by the Phase I CheckMate 012 study in advanced stage NSCLC after first-line platinum-based chemotherapy (NCT01454102) (Table 2). Of importance for correct data interpretation, the nivolumab plus bevacizumab arm included only patients with non-squamous histology, whereas the nivolumab monotherapy arm included patients with both squamous and non-squamous histology. Median PFS was 37.1 weeks in the combination arm compared to a median PFS of 16 weeks in squamous patients and 21.4 weeks in non-squamous patients in the nivolumab monotherapy arm. However, in contrast to PFS, no significant difference concerning OS was observed among the treatment groups. Also, the objective response rate (ORR) was similar between the nivolumab plus bevacizumab (8%) and nivolumab alone (10%) arms [31,32]. Based on the positive PFS results of this study, a second phase Ib study testing the safety and pharmacology of nivolumab in combination with nintedanib is going to be initiated soon (Available online: https://clinicaltrials.gov/, accessed on 29 October 2017).

Scientifically, it would be interesting to study, in sequential biopsies, the composition of immune cell type infiltration using new techniques such as single cell transcriptomics as it has recently been shown in a preclinical model that immune checkpoint blockade responses (anti-CLTA-4 or anti-PD-1) are driven by distinct cellular mechanisms [33]. In addition, to the best of our knowledge, the AA and immune-mediated molecular mechanisms, upon combinational treatment, have not yet been evaluated.

### 5.2. Clinical Studies Combining VEGF/VEGFR Targeting with Pembrolizumab

A multicenter phase I study (NCT02443324) is currently analysing the benefit of combinational treatment of ramucirumab with pembrolizumab in different tumor entities including NSCLC (Table 2). First interim analyses presented at the ESMO 2016 meeting revealed that 73% of patients (over all included cancer entities) experienced therapy-related adverse events (AE), with attention paid to lung cancer sub-analyses including 29 patients. A total of 7% of these NSCLC patients experienced grade 3–4 AEs including adrenal insufficiency, hyponatremia, delirium, infusion-related reaction. Interestingly, 30% of NSCLC patients had an objective response occurring in both histological subtypes (squamous, non-squamous) and in all PD-L1 groups (positive, negative). Median duration of treatment was 6.8 months or longer, median time to response was 1.45 months. However, probably due to the short investigation time, the median duration of response as well as median PFS have not yet been determined [34]. Updated analyses of this study are expected this year.

Furthermore, another Phase Ib trial investigating the impact of combined treatment of pembrolizumab and nintedanib in solid tumors including advanced NSCLC has been started and is currently recruiting participants (NCT02856425) (Table 2). Pembrolizumab plus the chemotherapeutic agents paclitaxel plus carboplatin with/without bevacizumab is also currently being evaluated in a phase I/II study in stage stage IIIb/IV NSCLC (NCT02039674) (Table 2).

### 5.3. Clinical Studies Combining Bevacizumab with Atezolizumab

In addition to pembrolizumab, the PD-L1 inhibitor atezolizumab is also evaluated in combination with carboplatin plus paclitaxel in a phase III study with or without the addition of bevacizumab in primary stage IV NSCLC (IMpower150) (NCT02366143) (Table 2). In addition, a phase 1b study concerning the safety and pharmacology of atezolizumab administered with bevacizumab in patients with advanced solid tumors including NSCLC is ongoing (NCT01633970) (Table 2).

## 6. Renal Cell Cancer (RCC)

RCC represents a common malignancy with 63,990 new estimated cases in 2017 in the US; 15% of newly diagnosed cancers are diagnosed in the primary metastatic RCC (mRCC) stage of disease [26]. In addition, 30% of initially localized diseases become metastatic, thus requiring efficient systemic treatment options [35].

In general, RCC is divided into different subtypes, among which clear cell RCC, arising from the proximal tubulus system, represents the most common subtype, representing 80% of RCCs [36,37]. The introduction of VEGFR-targeted TKIs has revolutionized the systemic treatment of RCC as the first approved TKI sorafenib was able to increase median PFS of mRCC patients from 2.8 months to 5.5 months [38,39]. Furthermore, the prolongation of the PFS by sorafenib translated into a significant OS benefit compared with a placebo (hazard ratio (HR) 0.72; 95% confidence interval (CI), 0.54 to 0.94; *p* = 0.02) [39].

Nevertheless, complete treatment response is observed in less than 1% of patients as most patients with initial response progress during AA therapy due to diverse resistance mechanisms. In the past year, the PD-1 inhibitor nivolumab has been Food and Drug Administration (FDA) approved in the second-line regime in mRCC, thereby re-launching immunotherapy as the standard treatment option [40]. However, long-term follow-up studies show that both treatment forms are limited due to the development of resistance mechanisms, which can possibly be overcome by combining both types of treatment.

## 7. Preclinical Concepts Combining AA and Immunotherapies in RCC

RCC represents a prototype of an AA-therapy sensitive tumor due to its unique molecular pathogenesis where inactivation of the Von Hippel–Lindau (VHL) tumor suppressor gene triggers a cascade of pro-angiogenic and thus tumor promoting events, resulting in an increase of growth factors such as VEGF advancing tumor growth, proliferation as well as migration of endothelial cells.

Already in 2008, it was shown for the first time that the TKI sunitinib reverses type-1 immune suppression and decreases Treg in RCC patients [41]. Sunitinib treatment decreases MDSC and Treg numbers in the tumor tissue and regulates the expression of negative co-stimulatory molecules such as CTLA-4 and PD-1 in both CD4 and CD8 T cells as well as PD-L1 expression on MDSC and plasmacytoid dendritic cells [42].

In 2009, it was shown, for the first time in 23 mRCC patients, that sunitinib reverses MDSC-mediated immunosuppression via reducing MDSC numbers as well as CD3^+^CD4^+^CD25^high+^Foxp3^+^ Tregs [43]. Recent data demonstrated that intra-tumoral CD8^+^ T cells increase following combination treatment of bevacizumab and atezolizumab in mRCC patients. Moreover, intra-tumoral major histocompatibility complex-1 (MHC-I), Th1 and T-effector cell markers and mostly the chemokine CX3CL1/fractalkine also increased [44].

## 8. Clinical Studies Combining AA and Immunotherapies in RCC

### 8.1. Clinical Studies Combining AA with Nivolumab

Since 2016, nivolumab has been approved for treatment of mRCC patients in the second-line setting after failure of primary AA treatment based on results from the phase III CheckMate 025 trial, showing increased OS in mRCC patients under nivolumab therapy (*n* = 406) compared to the mTOR inhibitor everolimus (*n* = 397) (25 vs. 19.6 months, respectively; HR 0.73; *p* = 0.002) [40]. Concerning its combination with VEGF inhibiting agents, the Phase I CheckMate 016 study currently investigates the safety and tolerability of combining nivolumab with either sunitinib or pazopanib (Table 3).

Initial data presented at the ESMO meeting in 2014, reported that 82% and 70% of patients in the sunitinib and pazopanib arm had grade 3 to 4 AEs respectively, leading to the consequence that the pazopanib treatment arm was closed because of several cases of high-grade liver toxicity. In contrast, the sunitinib arm was dose escalated to a higher nivolumab dose; moreover, treatment-naive patients were also included in the study.

Another Phase I study is testing the combination of nivolumab with cabozantinib, a dual VEGFR 2/c-MET inhibitor that has been approved for several months in second-line treatment of mRCC after demonstrating PFS and OS advantage over everolimus in the METEOR trial [45]. Preliminary data from 18 patients with the combination of cabozantinib plus nivolumab, alone or in combination with ipilimumab (NCT02496208) showed an ORR of 33%; however, only one patient with mRCC has been included in the study so far (all genitourinary cancers are allowed to be included in the study according to be study protocol (Available online: https://clinicaltrials.gov/, accessed on 29 October 2017) (Table 3).

### 8.2. Clinical Studies Combining AA with Pembrolizumab

Currently, safety and tolerability of pembrolizumab are being tested in combination, either with lenvatinib, pazopanib, bevacizumab or axitinib in three different phase I/II studies. Concerning the combination of pembrolizumab with the TKI axitinib (NCT02133742), therapy response has been shown in 35/52 patients while 11/52 patients had stable disease; moreover, analyses revealed that combination treatment is well-tolerated. In addition, the combination of prembroliumab plus axinitib showed therapy efficacy in the first-line treatment setting of mRCC patients. These initial promising results led to the initiation of a phase III trial of pembrolizumab with axitinib vs. sunitinib (NCT02853331). Thereby, 840 patients will be randomized in a 1:1 setting to either receive pembrolizumab every 3 weeks plus axitinib versus sunitinib once daily for 4 weeks followed by 2 weeks off. Treatment continues until progressive disease or unacceptable AE. Primary end points are to compare PFS and OS between treatment arms; secondary endpoints of the study include differences in ORR, duration of response, disease control rate, safety as well as patient-reported outcomes between the treatment arms (Table 3). 

Pembrolizumab plus levantinib, a multi-targeted VEGFR TKI with additional activity against FGFR, PDGFR, RET and c-KIT (approved by the FDA but not recommend by the EAU guidelines) is presently studied in a phase Ib/II study (NCT02501096) (Table 3). Results from the ESMO 2016 meeting, in which eight mRCC patients unable to receive any more standard therapies have been enrolled, revealed that the combination of these two substances is safe. Further analyses including a larger patient cohort are needed before drawing any conclusion about this kind of combination therapy.

Another phase III trial investigates the therapeutic efficacy of lenvatinib combined with everolimus or pembrolizumab vs. sunitinib alone in first-line treatment of patients with mRCC (NCT0281186) (Table 3). Patients are randomized 1:1:1 to receive lenvatinib 18 mg/day plus everolimus 5 mg/day, lenvatinib 20 mg/day plus pembrolizumab 200 mg every 3 weeks, or sunitib 50 mg/day until disease progression or unacceptable toxicities. The primary endpoint is to show the superiority of lenvatinib plus everolimus or lenvatinib plus pembrolizumab over single-agent sunitinib as a first-line treatment for advanced RCC in improving PFS. 

Pembrolizumab is also studied in combination with bevacizumab (NCT02348008) in a Phase I/IIb study. Treatment of 12 patients with pembrolizumab and bevacizumab showed no major AEs and was therefore assumed to be safe and will be further evaluated in a multicenter phase II study, which is planned to be initiated soon (Table 3) [46]. Lastly, a phase I/II study was designed to evaluate the safety and efficacy of pazopanib in combination with pembrolizumab [47]. Preliminary data from the ASCO 2017 showed that this combination is not feasible due to severe hepatotoxicity. Thus, sequential pazopanib followed by pazopanib plus pembrolizumab was assessed to improve tolerability. Although hepatotoxicity was not limiting, other severe AEs emerged requiring multiple dose modifications. As a consequence, poor tolerability does not support the initiation of the phase II part of this study [47].

### 8.3. Clinical Studies Combining AA with Atezolizumab

Atezolizumab is a fully humanized engineered monoclonal antibody of the immunoglobulin G1 (IgG1) isotype against the PD-L1 approved for solid tumors including lung, bladder and renal cancer.

Atezolizumab is currently being tested in combination with bevacizumab in a Phase II study after a successful Phase I study including 12 participants with no grade 4/5 side effects and 40% ORR including one complete remission. A first interim analysis was presented recently at the ASCO meeting in 2017, where atezolizumab was administered as monotherapy or in combination with bevacizumab compared to sunitinib in untreated RCC (IMmotion150 study, NCT01984242) [48] (Table 3). In PD-L1+ patients (54%), a PFS hazard ratio of 0.64 for atezolizumab plus bevazicumab vs. sunitinib alone was reached. A total of 78% of sunitinib and 60% of atezolizumab patients who progressed subsequently received atezolizumab plus bevacizumab and achieved ORRs of 28% and 24%, respectively. Therefore, the authors of the study concluded that atezolizumab plus bevazicumab exerts encouraging antitumor activity in first-line patients with PD-L1+ mRCC. In addition, preliminary activity in the second-line setting was demonstrated in patients who crossed over to atezolizumab plus bevazicumab, regardless of prior therapy. 

Based on these findings, a phase III open-label randomized trial has been initiated comparing the combination of bevacizumab plus atezolizumab with sunitinib monotherapy in which primary endpoints are PFS and OS (NCT02420821) (Table 3).

OX40 (CD134) is a tumor necrosis factor (TNF) receptor expressed primarily on activated CD4^+^ and CD8^+^ T cells, thereby transmitting a potent co-stimulatory signal. OXR0916 is a humanized IgG1 antibody against OX40. Preclinical models have shown that agonist anti-OX40 antibodies, such as MOXR0916, have a dual mechanism of action—when engaging OX40, the antibodies can attack the tumor through both co-stimulation of effector T cells and reduction of regulatory T cells. Recently, a phase I study investigating MOXR0916 plus atezolizumab vs. atezolizumab plus MOXR0916 plus bevacizumab has been initiated in solid tumors including mRCC patients (NCT02410512). Currently, the study is recruiting participants (Table 3).

## 9. Further Ongoing Studies Combining Immunotherapy and AA Therapy in RCC

JAVELIN Renal 100 (NCT02493751), a phase I study, is evaluating the safety and clinical activity of avelumab plus axitinib in treatment-naive patients with RCC [49] (Table 3). Data from the ASCO 2017 meeting showed that 51 of 55 enrolled patients (92.7%) had an avelumab-related adverse event with the therapy combination, among which the most common were diarrhea (52.7%), hypertension (45.5%), dysphonia (43.6%), and fatigue (43.6%). Also, two deaths occurred in the study during the reporting period, one due to progression and one related to both treatments (myocarditis). Despite the side effects, confirmed ORR was 54.5% (95% CI 40.6–68.0) based on two complete responses and 28 partial responses. 

Based on the data of JAVELIN Renal 100, JAVELIN Renal 101, a randomized, multicenter, phase III study (NCT02684006) comparing avelumab (fully human IgG1 anti–PD-L1 antibody) plus axitinib vs. sunitinib in treatment-naive RCC patients has been initiated—the primary objective is to demonstrate the superiority of avelumab in combination with axitinib compared to sunitinib in PFS (Table 3). The trial has been active for several months at 103 sites across 12 countries and already more than 40% of patients have been enrolled since February 2017. 

## 10. Conclusions

To summarize, both NSCLC and mRCC represent angiogenesis-dependent neoplasms and immunogenic tumor entities highly amendable for both AA and immunogenic therapies such as checkpoint inhibition. However, both treatment options are associated with resistance mechanisms leading to primary or secondary treatment failure and ultimately tumor progression and death. 

Preclinical models provide a clear rationale for the combination of anti-VEGF agents together with immune checkpoint blockade by exerting potent synergistic anti-tumor activity. Consequently, numerous clinical studies evaluating this novel therapy combination in diverse tumor entities focusing on NSCLC and RCC have been initiated, whose preliminary data presented at the ASCO or the ESMO meetings are highly promising with tolerable side effects. Initial phase I/II data reported the superiority of combined treatment compared to monotherapy; nevertheless, these data have to be interpreted with caution, as data of phase III studies have to be awaited before drawing final conclusions. In addition, to date, comparison to the approved monotherapy studies seems to be difficult as therapy algorithms have changed over time, and only head to head comparisons can shed light on these open issues. Thus, in the next 3–5 years, we expect practice-changing results from ongoing studies of immune-oncology in combination with AA agents also in other malignancies such as colorectal cancer and melanoma, in addition to both entities described in this review.

## Figures and Tables

**Table 1:** Overview of target agents used as combination strategies in NSCLC and RCC.

Drug	Substance Type	Molecular Target
Avelumab	mAB	PD-L1
Atezolizumab	mAB	PD-L1
Axitinib	TKI	VEGFR 1–3, PDGFR, c-KIT
Bevacizumab	mAB	VEGF-A
Cabozantinib	TKI	VEGFR 2, c-MET
Cetuximab	mAB	EGFR
Durvalumab	mAB	PD-L1, CD80
Lenvatinib	TKI	VEGFR 1–3, FGFR, PDGFR, RET, c-KIT
Nivolumab	mAB	PD-1
Nintedanib	TKI	VEGFR 1–3, FGFR 1–3, PDGFR
Pazopanib	TKI	VEGFR 1–3, PDGFR, c-KIT
Pembrolizumab	mAB	PD-1
Ramucirumab	mAB	VEGFR 2
Sunitinib	TKI	PDGFR, VEGFR 1–3, c-KIT, FLT

TKI, tyrosine kinase inhibitor, mAB, monoclonal antibody; PD-L1, programed death ligand-1; VEGFR, vascular endothelial growth factor receptor; FGFR, fibroblast growth factor receptor, PDGFR, platelet-derived growth factor receptor, c-MET, tyrosine-protein kinase Met, RET, ret proto-oncogene, c-KIT, KIT proto-oncogene receptor tyrosine kinase, PD-1, programed cell death protein-1.

**Table 2:** Ongoing clinical studies combining AA and immunotherapies in NSCLC.

Study Name	Targeting Agents	Comparison	Phase	Primary Endpoint	Therapy Setting	Status *	NCT Number
CheckMate 012	bevacizumab + nivolumab	bevacizumab	I	PFS, OS	First line maintenance	Active Not recruiting	NCT01454102
N.a.	ramucirumab + pembrolizumab	ramucirumab	I	PFS, OS	Inoperable patients	Recruiting	NCT02443324
IMpower150	Atezolizumab + bevazicumab carboplatin + paclitaxel	Atezolizumab + carboplatin + paclitaxel	III	PFS, OS	First line (Stage IV)	Recruiting	NCT02366143
N.a.	atezolizumab + bevacizumab	/	Ib	Safety	First line	Active Not recruiting	NCT01633970
N.a.	pembrolizumab + nintedanib	/	Ib	MTD of nintedanib, Safety	First line	Recruiting	NCT02856425
N.a.	nivolumab + nintedanib	/	Ib	Safety	Second line	Not recruiting	TBA
N.a.	pembrolizumab + paclitacel + bevazicumab	Pembrolizumab + paclitacel	I/II	Clinical efficacy and safety	First line (Stage IIIB/IV)	Recruiting	NCT02039674

* status according to https://clinicaltrials.gov/, accessed on 8 July 2017. OS, overall survival; PFS, progression free survival; N.a., not assessed; MTD, maxium tolerated dose; TBA, to be announced; NCT, clinicaltrials.gov registry number.

**Table 3:** Ongoing clinical studies combining AA and immunotherapies in RCC.

Study Name	Targeting Agents	Comparison	Phase	Primary Endpoint	Therapy Setting	Status *	NCT Number
WO29637	atezolizumab + bevacizumab	sunitinib	III	PFS, OS	First line	Active Not recruiting	NCT02420821
JAVELIN Renal 101	avelumab + axitinib	sunitinib	III	PFS	First line	Recruiting	NCT02684006
JAVELIN Renal 100	avelumab + axitinib	/	I	MTD	First line	Active Not recruiting	NCT02493751
200249	pembrolizumab + pazopanib	monotherapy	II	Clinical efficacy and safety	First line	Active Not recruiting	NCT02014636
KEYNOTE-426	pembrolizumab + axitinib	sunitinib	III	PFS, OS	First line	Recruiting	NCT02853331
E7080-G000-307	lenvatinib + pembrolizumab or everolimus	sunitinib	III	PFS	First line	Recruiting	NCT02811861
N.a.	pembrolizumab + axitinib	/	Ib	Safety, treatment efficacy	First line	Active	NCT02133742
N.a.	pembrolizumab + lenvatinib	/	Ib/II	MTD, ORR	No standard therapies anymore available	Active	NCT02501096
BTCRC-GU14-003	pembrolizumab + bevacizumab	/	Ib/II	Safety, efficacy tolerability	At least second line	Active Not recruiting	NCT02348008
N.a.	atezolizumab + MOXR0916 + bevacizumab	atezolizumab + MOXR0916	I	Dose limiting toxicities Side effects	Any	Recruiting	NCT02410512
CheckMate 016	nivolumab + sunitinib or pazopanib or ipilimumab	nivolumab	I	Safety tolerability	First line Second line	Recruiting	NCT01472081
N.a.	cabozantinib + nivolumab	cabozantinib + nivolumab + ipilimumab	I	Safety tolerability	No standard therapies anymore available	Recruiting	NCT02496208
IMmotion150	atezolizumab +/−bevacizumab	sunitinib	II	PFS	First line	Active Not recruiting	NCT01984242

* status according to https://clinicaltrials.gov/, accessed on 8 July 2017. ORR, objective response rate.
